# Racism during clinical placement, the perpetrators, impact, advocating and reporting

**DOI:** 10.1177/09697330251317675

**Published:** 2025-02-07

**Authors:** Hila Ariela Dafny, Nicole Snaith, Christine McCloud, Nasreena Waheed, Paul Cooper, Stephanie Champion

**Affiliations:** 64767 Flinders University; 1067 University of South Australia; 64767 Flinders University; College of Nursing and Health Sciences

**Keywords:** Ethics education, incivility, nursing, qualitative research, racism, workplace violence

## Abstract

**Background:** The experience of racism in healthcare is particularly challenging to address due to misunderstandings of the definition, the complex interplay of other potential discriminations and, at some level, the denial that it occurs. Limited studies have reported racism as an aspect of workplace violence toward nurses and nursing students from both patients and staff.

**Research aims:** To understand nursing students’ experience of unethical behaviour, including racism during clinical placement, the perpetrators, impacts, advocating and reporting.

**Research design:** An interpretive, qualitative design was used, and 15 nursing students were interviewed using semi-structured interview guides. The interview recordings were transcribed and thematically analysed.

**Participants and research context:** Nursing students voluntarily participated and completed the interviews for this study from one undergraduate nursing student cohort in metropolitan South Australia.

**Ethical considerations:** This study received ethical approval from the University Social and Behavioural Research Ethics Committee.

**Findings/results:** The two major themes with subthemes of the findings include (1) The multi-faceted student nurse experience of racism: sub themes – racism from patients to nurses, from nurses to nursing students’ and racism towards patients. (2) The pervasive influence and limited reporting of racism by nursing students: sub themes-feeling disempowered, and barriers to reporting racism.

**Conclusions:** The findings of this study highlight the registered nurse students’ experience of racism in various forms within the clinical environment and the significant negative impact it has on RNS during placements. This evidence calls for systemic changes to create a more inclusive and supportive environment for all RNS.

## Introduction

Racism has been defined as ‘an ideological construct that assigns a certain race and/or ethnic group to a position of power over others on the basis of physical and cultural attributes, as well as economic wealth, involving hierarchical relations where the “superior” race exercises domination and control over other’,^1^ (p. 4). The United Nations Educational, Scientific and Cultural Organization (UNESCO) 1978 Declaration on Race and Racial Prejudice states that ‘racism includes racist ideologies, prejudiced attitudes, discriminatory behaviour, structural arrangements and institutionalized practices resulting in racial inequality as well as the fallacious notion that discriminatory relations between groups are morally and scientifically justifiable; it is reflected in discriminatory provisions in legislation or regulations and discriminatory practices as well as in anti-social beliefs and acts’,^2^ (p. 62–63).

The experience of racism in healthcare is particularly challenging to address due to misunderstandings of the definition, the complex interplay of other potential discriminations and, at some level, the denial that it occurs.^3^ A study involving five focus groups (*n* = 31) with South Australian maternal, child and family nurses found misunderstandings of what racism is and that it is often confused with inequalities based on gender and poverty.^4^ Notably, described racism was viewed as intersecting with other discriminations, specifically gender and student status, which further complicates actions toward acknowledging, reporting and addressing this significant issue.^5^ Adding to the challenge to address racism, an integrated review of the literature on race and racism in nursing reported a common theme of ‘denial and silencing of racism’ within the profession.^6^ Furthermore, racism is often considered a difficult issue to discuss due to evoking feelings of anxiety and discomfort, shame, anger and guilt. The fear of saying the wrong thing or being misunderstood is a significant barrier.^6^ Australian nursing as a white dominant profession, may further perpetuate this silencing, and avoidance of acknowledging and discussing racism by minority ethnic health care providers^7^ It is evident from the literature that the experience of racism could easily be dismissed, minimised or challenged by those in a position of authority.

Racism in healthcare is well-acknowledged in the literature but predominantly focused on the adverse experiences and poorer health outcomes experienced by patients.^8^ The research in this area is also dominated by the USA and UK, with little literature on the Australian experience of racism in healthcare. A recent scoping review of racism in health care including research predominantly from the USA (67%), but also the UK (7%), Canada (7%), Australia (5%) and New Zealand (3%), found a common theme of a lack of organisational support for staff experiencing racism, a range of staff negative stereotypes of ethnic minority groups and the culture of normalising and not discussing racism in healthcare.^8^ In the Australian context, the experience of institutional racism and inequalities faced by Aboriginal and Torres Strait Islander people in the healthcare system, from medical education to funding, policy and clinical settings, is emphasised.^7^ The authors propose that this systemic discrimination within the Australian healthcare system stems from the historical context and treatment of First Nations people in Australia.

All of the contributors to this paper are or have been university educators for registered nurses RNS and have heard the many stories of racism experiences during clinical placement that students discuss freely in class. Combined with past research evidence from the literature and the findings of this research groups previous work on WPV, this anecdotal evidence became the impetus for this research project.

## Background

The education of registered nurses is now almost exclusively the purview of the higher education sector which is responsive to both global and university specific student recruitment trends.^9^ In the early years of baccalaureate nurse training, universities aimed to provide a quality vocational pathway to meet domestic needs with few international students recruited.^10^ However, recent higher education trends have seen a rapid globalisation in the knowledge economy, where higher education student recruitment aims to meet the demands of a global rather than domestic market.^10^ In Health care education, the result of this trend is a registered student nurse (RNS) cohort that is culturally diverse from the dominant domestic culture and where hands on learning occurs in an environment where work-place violence including racism is rampant.^5^ Adding to the complexity of this cohort in Australia, is recognition of the health inequities between dominant white Australian population and First Nation peoples and the active recruitment of First Nations health care students into the university system.^11^ This recruitment strategy adds a further cultural diversity to the RNS cohort whose experiences of racism in the clinical environment have been identified but not well understood.^5^

Racism experienced by nurses and registered nurse students (RNS) during work or clinical placement is considered by the limited literature as a form of workplace violence (WPV).^12–14^ Only a few studies^15,16^ reported on nursing students being subjected to a ‘racist remark’ rather than other less overt experiences of racism and classified it under ‘non-violent’ bullying behaviour. This may not be a true reflection of the levels of racism experienced by RNS and the lack of understanding of what constitutes racism may be a barrier to addressing this significant issue. In a cross-sectional survey of Australian undergraduate RNS (*n* = 888), 19% reported experiencing a racist remark during their clinical placement.^15^ Similarly, a cross-sectional survey of UK undergraduate RNS (*n* = 657) found that nearly 10% of students had experienced a racist remark during their clinical placement^10^ and racism from patients to students was a key theme in another UK study.^13^ A more recent study in the UK reported persistent racism within the health and higher education sector toward RNS and qualified nurses.^5^ Reports of racism toward international students or those from a non-English speaking background were a consistent theme in the literature.^14,17^ Negative comments from qualified nurses on students’ English language skills and abilities as nurses, as well as being highly critical of their work, were witnessed in a survey of Australian RNS (*n* = 884).^17^ One student described witnessing racist remarks about Aboriginal and Torres Strait Islander people and patients during handover, in the staff room, in educational sessions and in front of patients during their 3rd year placement.^17^ Similarly, a cross-sectional survey of New Zealand students (*n* = 296) showed consistent themes of racism toward international students and racist comments toward or about Māori students from clinical staff.^14^

Limited research has examined the impact of racism on RNS, but most studies suggest that students may feel isolated and unsupported.^17^ Students in a study undertaken in the UK exploring Black African-Caribbean RNS experiences of racism in clinical and education settings reported inequity in accessing learning opportunities further perpetuated by staying silent for fear of failing or being seen as a troublemaker or ‘playing the race card’, high levels of stress and uncertainty in their future as a nurse, social withdrawal and isolation as coping strategies, loss of self-confidence and trust in the nursing education and clinical placement settings.^18^ Detrimental effects on the physical, mental and emotional well-being of students experiencing racism are highly likely, which may further contribute to student nurses leaving their studies or the nursing profession.

A scoping review of racism in healthcare found that many studies reported that healthcare staff find it difficult to discuss racism and accept racism as part of the healthcare system.^8^ Miller and Nambier-Greenwood claim that the nursing profession and nurse educators are reluctant to address racism.^18^ The very notion of racism conflicts with the core of nursing values; admitting that it is a reality for students is perhaps taking some level of accountability. This may be one of the major challenges in addressing racism toward RNS. This finding is consistent with research on racism in healthcare in general with two recent reviews citing the common theme of ‘silencing’ and normalising of racism experienced by nurses^6^ and other healthcare staff.^8^ This paper explores the clinical learning experiences of a culturally diverse RNS cohort in an environment where racism is endemic.^8^

### Research aims

The study aims to examine nursing students’ experiences of unethical behaviour, including racism during clinical placement, the perpetrators, impacts, advocating and reporting.

## Research design

An interpretive phenomenological qualitative design was employed in this study to examine participants’ lived experiences of workplace violence including racism and its effect on their lives. This methodology was selected to generate rich, detailed data that provides potential for an in-depth understanding of participants’ lived experiences.

### Theoretical framework

To explore the lived experiences of workplace violence, this study adopted an interpretative phenomenological approach guided by a philosophical position where understanding of the lived experiences is inherently an interpretive process.^19^ This methodology is particularly suited for investigating participants lived experiences through hermeneutic interpretive processes that seeks to understand and connect the individual human experiences of a phenomenon.^19^

### Study setting and recruitment

Participants for this study were recruited using purposive sampling from a single university in metropolitan Adelaide, South Australia, where the researchers are employed. Recruitment was conducted via email invitations and campus posters. Interested individuals who contacted the researchers were provided with a detailed explanation of the study, and informed consent was obtained prior to participation.

Sample size was not a primary concern, as qualitative research often achieves saturation with small sample sizes, particularly in homogeneous populations with narrowly focused objectives (e.g. 9–17 interviews).^20^ In such studies, sample sizes may range from a single participant to as many as needed to reach saturation,^21^ which was the approach adopted in this research.

### Inclusion and exclusion criteria

The inclusion criteria required participants to be Bachelor of Nursing students from the specified university who had completed a clinical placement and consented to participate in the study. Students who had not completed a clinical placement or were enrolled in other programs were excluded.

### Data collection

Participants who consented to interviews were given the option to choose their preferred format, either face-to-face or online via Microsoft Teams. To ensure comfort and privacy, all interviews were conducted outside of the clinical setting. Prior to the interviews, participants completed a demographic information sheet and were reassured of their right to withdraw consent at any time without any negative consequences.

Individual semi-structured interviews were conducted between September and November 2022, guided by an interview protocol developed by the research team based on a literature review. Interviewers were provided with the protocol outlining core questions, but the semi-structured format allowed for flexibility to explore different issues that arose during individual interviews.

Open-ended questions were used to gain a deeper understanding of participants’ experiences and perceptions of workplace violence (WPV). For example, participants were asked, ‘Have you ever experienced or witnessed workplace violence while you were on clinical placement?’ Follow-up questions included, ‘Would you like to share your experience?’ and ‘Can you provide examples?’ These questions encouraged narrative responses, allowing participants to freely express their thoughts and enabling the collection of a rich data set.

After the first five interviews, the research team met to evaluate whether participants were interpreting the questions consistently and to refine the approach if needed. Each interview lasted approximately 60 min and data collection continued until thematic saturation was reached.

### Data analysis

During data collection, interviews were audio-recorded, transcribed verbatim and analysed using Braun and Clarke’s thematic analysis approach.^22^ The transcripts were read and re-read to ensure familiarity with the data, and initial impressions were documented. Common ideas were identified and grouped into codes, which were subsequently organized into overarching themes. The initial coding process was facilitated by QSR NVivo 12 software.

### Ethical considerations

The study was approved by the Human Research Ethics Committee (number 5497) of the researchers’ affiliated university. Participation was entirely voluntary, with participants free to withdraw at any time without consequences. Informed consent was obtained for both participation and the dissemination of findings.

Confidentiality was safeguarded by de-identifying participant information, limiting data access to the research team, and deleting recordings after transcription. De-identified transcripts were securely stored on the University password protected R drive and will be retained for 5 years following the project’s completion.

To prevent potential conflicts of interest, research team members involved in clinical placement topics were excluded from conducting interviews. Interviews were facilitated by three female researchers, two of whom spoke English as a second language, and two were born outside of Australia.

The study prioritized participants’ mental and emotional well-being. At the start of each interview, participants were informed they could stop the session at any time if they felt distressed. Additionally, they were provided with a list of support services for further assistance if needed.

### Rigour and reflexivity

Rigour was upheld by adhering strictly to the research process, with the research team conducting regular meetings to ensure alignment, clarify interpretations, and maintain consistency throughout the study. The initial thematic analysis was carried out by two team members and subsequently reviewed and refined through a collaborative feedback process involving the entire team to ensure accuracy and reliability. Investigator triangulation, as outlined by Polit and Beck, was integral to the analysis, reducing bias and minimizing the risk of subjective or idiosyncratic interpretations.^19^

## Findings

### Demographic profile

All participants were full-time RNS; eight were in their second year, and seven were in their third year (after completion of at least one clinical placement) (Table 1). Registered nurse students ranged from 22 to 60 years (average 35 years) with four students preferring not to mention their age, 12 females and 3 males. The cultural background or origin of the RNS was diverse, with nine RNS internal and English as their second language and six Australians (non-First nations). The international RNS were from six countries including, Nepali, Vietnam, Sri Lankan, Iranian, India and China. All RNS had prior employment before commencing nursing, except one 22-year 3rd-year Australian student who was a high school student before commencing nursing. The RNS’s previous employment included, working in disability as a support worker, pizza maker, software tester, nurse (in their home country), manager, early childhood educator, manager, care assistant, pharmacology student, hospitality, practice manager at a GP clinic, retail manager and oral health therapist.

**Table 1.** The demographic profile of registered nurse students.

Pseudo names	Age	Gender	Cultural background	Year level	Full time/part time
Participant 1	Preferred not to say	M	Indian	2nd year	Full time
Participant 2	24	F	Indian	3rd year	Full time
Participant 3	29	F	Nepali	3rd year	Full time
Participant 4	34	F	Chinese	2nd year	Full time
Participant 5	Preferred not to say	F	Sri Lankan	2nd year	Full time
Participant 6	30	M	Australian	2nd year	Full time
Participant 7	22	M	Australian	3rd year	Full time
Participant 8	46	F	Iranian	3rd year	Full time
Participant 9	30	F	Australian	3rd year	Full time
Participant 10	Preferred not to say	F	Vietnamese / Chinese	2nd year	Full time
Participant 11	24	F	Australian	2nd year	Full time
Participant 12	39	F	Chinese	2nd year	Full time
Participant 13	30	F	Australian	2nd year	Full time
Participant 14	60	F	Australian	3rd year	Full time
Participant 15	Preferred not to say	F	Chinese	3rd year	Full time

### Themes and sub-themes

Registered nurse students their experiences of racism and its impact on them. Two main themes and five sub-themes were identified (Table 2). The main themes included (1) The multi-faceted RNS experience of racism and (2) The pervasive influence and limited reporting of racism on RNS. The sub-themes of theme one included (1) Racism from patients, (2) Nurses’ to nursing students’ racism and (3) Racism towards patients. The sub-themes of main theme two were (1) Feeling disempowered and (2) Barriers to reporting racism. The length of the quotes reported in this study reflect the difficulties of describing racist incidents.

**Table 2.** RNS experience and impact of racism themes and sub-themes.

Themes	Sub-themes
The multi-faceted RNS experiences of racism	Racism from patients
	Nurses to nursing students’ racism
	Racism towards patients
The pervasive influence and limited reporting of racism on RNS	Feeling disempowered
	Barriers to reporting racism

### Experience of racism during placement

The first theme was the multi-faceted experience of racism during placement and includes three sub-themes: (1) Racism from patients, (2) Nurses to nursing students’ racism and (3) Racism towards patients. This theme captures the broad-base nature of events and perpetrators of racism experienced and witnessed by RNS.

### Racism from patients towards nursing students

Within the interview cohort, many of the international RNS reported they experienced racism from patients lashing out wildly in the aged care facility, and that racism came in the forms of verbal violence, cursing and shouting at them or non-verbal violence and ignoring them.

A RNS described an experience they had when they prepared a coffee for a patient. The patient was unhappy with the coffee and reacted by shouting and cursing at the RNS. The patients’ response to the incorrect coffee order was angry and aggressive in behaviour underlying a perceived racially based limitation of the student. The patients’ perception of the international RNS assumed limited and an incorrect capacity to communicate in English thus displaying inherent racism.

*So, he asked me if I can make a coffee for him*. … *I bring the coffee to him. He was shouting at me*. *I said, what happened? You asked for three sugar*…. *[He said] “don’t you understand English, just a coffee with three sugars*.*” He was shouting at me as well. I say, oh sorry, I made the wrong coffee … And he just keep shouting … then he say (curse words)*.*” PARTICIPANT 12*

International RNS found there was an assumption amongst patients that RNS who spoke languages other than English were not able to provide quality care. This assumption was linked by patients with an RNS’ capacity to provide quality care and to communicate effectively.

*[Patients] say that, oh, you are not – you are not English speaker, you are not good at speaking English, you are not suitable to look after me. Some people will think in that way. PARTICIPANT11*

A RNS [who was an international student] shared their perspective from their placement in an aged care facility. Their reported experiences demonstrated unconscious biases held by patients towards RNS despite receiving timely and supportive care

*…Staff is here to help 24/7, anything you want, you press the call bell, and staff comes and help you… The words they use sometimes might really hit you somewhere you know the words they use, sometimes they’re racist, sometimes they use a lot of mean words you know. PARTICIPANT14*

### Nurses’ racism towards nursing students

Some RNS experience racism during their clinical placement from staff nurses who were from culturally diverse backgrounds and who spoke together in another language rather than English, often a language that the RNS could not understand. The RNS felt uncomfortable and as if they were being talked about and the behaviour by these staff was disrespectful and inherently racist. Other staff displayed incivility towards RNS, often failing to be inclusive and to assist students with their learning through responding appropriately to questions asked by RNS.

*…most of the time they’re speaking their own language, not in English but in other language in front of me and they are, I feel they are talking about me, and they are telling something back to me like that. PARTICIPANT15*

*‘[The staff] I was working with; I asked a question, and they just totally ignored me and kept walking.’ PARTICIPANT2*

Registered nurse students reported incidences of racism and favouritism of staff nurses towards RNS from the same background and countries as the staff in comparison to those from other countries and cultures. When this occurred the RNS sought clarification from students who understood the language and it was evident that the behaviour of the staff nurse was uncivil and lacked professionalism and respect toward RNS

*I did talk to them and luckily two of the girls were actually from the country the nurses were from. So they told me they were gossiping about you. Yeah, but they were quite shocked. I think they were close with those nurses because they are from the same country. So I think that’s a bit of racism as well because those nurses were good to the students from the one country and not the outsiders…. I remember when I told those two girls that has happened, and they were like really shocked that that particular nurse could do it, so we were really like I could see like they were close with the nurse. PARTICIPANT12*

### Racism towards patients

Not all racism events witnessed by RNS were directed at themselves with instances of poor behaviour and poor care by staff nurses directed to patients from culturally diverse backgrounds reported. When these events occurred RNS stated that they felt ignored, and were witnessed to racism and a lack of empathy towards patients, that resulted in patients not receiving the care they requested, or delays in addressing health issues. Furthermore, when trying to advocate for patients who were from culturally diverse backgrounds the RNS were ignored or recipients of the staff nurse anger which led to feelings of disempowerment. Racially based assumptions and motives for Australian first nations people were made by staff nurses towards patients and witnessed by RNS.

*“…I actually went very far out of my way to make sure that a indigenous woman who was on the ward was being understood and being listened to… she kept saying “This hurts, I’m in pain” and she was speaking in her language and I kept asking her “What’s going on, how can I help you” and I was asking her “What does that mean in your language, can you please tell me what does pain mean in your language?” She spoke Pitjantjatjara and I was really trying to communicate with her and talk to her, I asked if maybe she needs a translator or an Aboriginal health worker to help to figure out what’s going on, what’s causing her more pain. I mean they [the staff nurses] just kept saying “She’s an alcoholic, she was an alcoholic and now she’s in hospital for because she’s having this issue” and I kept saying “No I think there’s something not right, she’s really complaining” and as it turned out they had stuffed up and they hadn’t given her Albumin which she really actually desperately needed … she was actually very-very sick and I said something about it to the doctor. I said “I’m so sorry, I said something about this and no-one listened to me” and he and the nurses heard me say that to the doctor and then they got pissed off at me for it. So that was just another situation in itself, I really tried to advocate for this patient and just nothing happened’” PARTICIPANT3*

*“…we had a couple of patients on the ward who were First Nation peoples and – in handover it was, they were just drama queens [by staff nurses]. They were just using the system to get more housing – that sort of thing” PARTICIPANT2*

In the first theme of this study, RNS described their experiences of racism during their clinical placements from a broad base of perpetrators including patients, nurses and staff toward patients. Included in their experiences was racism toward First Nations patients and how their limitations as RNS in trying to advocate for these patients. Their stories highlighted the multifaceted and pervasive nature of racism witnessed or experienced by RNS. In the second theme, RNS described the ways racism impacted on their feelings and capacity to advocate for patients, and how these experiences could be linked to their own personal circumstances and experiences. Barriers to reporting of racism is also explored in the second theme.

### The impact of racism on nursing students

When RNS reported experiences or witnessing of racism, the events they described sparked internalised feelings that were often linked to their own past negative experiences. This linking of racism events during clinical placement and their own histories were often self-interpreted as powerlessness, fear and at times anger and became the sub theme of this second major theme. The second theme of this study explores the individual consequences of racism and the barriers to reporting racism.

### Feelings of disempowerment

Witnessing or experiencing racism in the placements triggered personal negative experiences, and some RNS felt powerless, disempowered, under pressure, and feared failing their placement. Others RNS were unhappy but tried to control themselves and continue to practice professionally, providing the best care they could offer to the patients.

*“I just tried to behave – I mean, I tried to perform and practice as my professional behaviour, because I learnt from the unit we should not use some judgement or attitude. I don’t, I feel unhappy about that, but I tried to control myself and just do my normal nursing practice.” PARTICIPANT11*

*“with the racism in regard to domestic violence and things… that triggered some past experiences for myself. But it also made me feel quite powerless… I really just want to scoop everyone up and run. Basically, it disempowered me, and I thought, what do I do? - who do I turn to? I’m still a student. I don’t want to muck up this placement. You have all those kinds of pressures coming at you as well.” PARTICIPANT2*

*“So I just I felt so annoyed and that’s why I spoke to the doctor, I said something about that because I did, I said something about it and no-one did anything and I felt embarrassed that I was looking after this lady and nothing happened. Because even as a student I expect that people would still advocate for their patients no matter what.” PARTICIPANT 3*

*“what I actually did was made sure I spent time with these people, found out what they needed… you get the best care, you get whole care. You get everything from your file related to what’s happening to you today, and things aren’t ignored.” PARTICIPANT2*

### Barriers to reporting racism

Despite a few RNS who advocated for others, most RNS did not report racism. Registered nurse students felt unable to report the racism and violence they have experienced for several reasons, including a fear that the nurse would think the RNS were not performing well. Registered nurse students thought reporting racism would give the impression they were unable to do their job, or they were concerned the response would seem disproportionate and that they would be perceived to be making a big fuss and getting someone in trouble for being rude.

*“So, I feel like if I report this, that they think my performance not good, because we require the feedback form from the nurse, that’s why I didn’t dare to say anything about this.” PARTICIPANT 12*

*“I just saw signs on the ward, signs on the wall, signs that the violence is not allowed, if it happened, then we call the police. I just feel like, okay, this guy, he’s being so rude and probably being racist, but I can’t just because of this, and call the police, right, it’s let the police come, they think you are too ridiculous.” PARTICIPANT11*

*… But I didn’t want to go out of my way to say something if it was wrong, I didn’t want to overly, I didn’t want people to get pissed off at me because I was trying to tell them that what they were doing was wrong, when they’ve been practising for 10 years. I just kept saying “Maybe we can check again,” I felt like I was trying to help and just no-one listened so yeah.” PARTICIPANT3*

## Discussion

This study aimed to examine nursing students’ experiences of racism during clinical placement, describing the perpetrators of racist behaviours, the impact of these experiences RNS, and RNS reporting of racism. The RNS in this investigation reported that both RNS and patients experienced episodes of racist behaviour while working and receiving care in healthcare settings, with perpetrators of this violence including patients and nursing staff. This experience of discrimination while on placement has been previously identified in Australian studies, finding RNS who perceive themselves to be different from the norm due to their age, sex, ethnicity, language, religion or sexual orientation, among other reasons, are more likely to experience prejudice or discrimination from staff and patients while on placement in health care settings.^23^ This finding concurs with the findings of a study completed in Sweden where Odzalovic et al. outlined the routine experience of racism with people names appearing ‘Non-Swedish’ and the dismissal of experience that would then occur.^24^ Odzalovic e al. continue on with explaining that students felt non-discussion of racism to be a characteristic of the nursing role which is a demonstration of the global nature of this racism.^24^ A study conducted by Zhou et al., in 2017 also demonstrated this ‘othering’ of racism with nurses educated in China and working in Australia outlining the experiences of racism they experienced, from differences being seen as incompetence to othering in social gatherings and exclusion due to cultural differences, suggesting that a sense of being alongside rather than with was the experience these nurses had.^25^

The examples of racism RNS provided demonstrated their struggles to define instances of racism due to the complexities of language and the way racism intersects with other forms of discrimination attributable to their gender and inexperience.^5^ Registered nurse students reported feeling excluded when other students and staff communicated in languages beyond their proficiency and felt helpless to intervene upon witnessing staff members stereotyping patients, negatively impacting patient care. These experiences made RNS disheartened about their studies and future career prospects. As with other forms of violence, RNS in this study were reluctant to report incidents involving racist language used against them by staff or patients. They perceived these behaviours as too subtle to classify, feared that the consequences of reporting would be disproportionate to the offences, and were concerned that making a report might be seen as an overreaction, potentially jeopardising their positions in clinical placement.

Racism is not unique to Australia/health care settings, and early examples of racism in nursing were poorly explored. Hantke et al. highlighted that from an early article written by Vaughan (1997) asking if racism was present in nursing through to studies in 2020, few studies critically addressed the issue of racism in health care.^26^ This lack of literature highlights the pervasive nature of racism in nursing and the underreporting that can be identified from this dearth of studies. International studies have demonstrated that healthcare workers often struggle to recognise the presence of racism, or address racism within healthcare systems as it contradicts the fundamental values of health professionals.^8,18^ The critical discussion of racism in nursing not being held has created a culture globally where students who are experiencing racism are feeling silenced in their experiences.^27^ Increased global immigration and population movements have increased the experiences of this issue, reinforcing that it is not an issue faced by any one nation alone.^27^

Saadi et al. interviewed nurses who reported that they needed to suppress parts of their identity to ‘embody professionalism’; these included aspects of appearance, including hairstyles.^28^ Saadi et al. also highlighted the issues of racism impacting retention, even of those qualified to leave the role, suggesting this would be more significant for those still students, with less invested in their careers.^28^ This potential attrition can be seen as stemming from unsupportive management who are not explicitly admitting to a racism issue in the workplace, some of this being shown in neutrality, staying silent when witnessing racism in the workplace^28^ and concurred with the findings of this study.

If racist behaviours cannot be adequately confronted, the cycle of workplace violence will continue.^29^ The nursing students exposed to racism will continue to believe they need to adjust to the workplace culture rather than call out discriminatory language and practices. Registered nurse students were already apathetic regarding harmful behaviours from patients, either dismissing this abuse as simply part of the job^12^ or concluding that even if they speak up, nothing will be done, so there is no point in complaining.^5^ It is reasonable for RNS to feel safe in the workplace; however, evidence has displayed the imbalance of staffing, showing that in 2015, Indigenous Australians only made up 1.1% of all midwives and nurses registered to practice, despite being 3% of the overall population.^30^ This imbalance stems from historical racism of segregation through to a tertiary education system with a very heavy European bias that can be seen to be exclusionary of some cultural groups.^31^ A recent review examining the discourse on racism and colonialism in western nursing scholarship found racial prejudices are systematically and structurally embedded into education and health care systems, contributing to ongoing injustice and disparities in outcomes for marginalised groups.^6^ This limitation of equity is extended further by Taylor et al., who outlined that when students do not feel supported or understood within the workforce, this increases loneliness, isolation and stress.^30^ Stereotyping and racial prejudices were reported in an Australian study of RNS as some of the violence experienced on placement; RNS reported that this form of racism was not investigated with minimal consequences for perpetrators.^32^

While beyond the scope of this paper, the interviews could be utilised in an investigation of the intersection between racism and gender. Twelve of the 15 interviewees (80%) in this study identified as female, and of those eight came from non-Australian cultural backgrounds. This is representative of the gender division of the nursing workforce in Australia^33^ but more diverse in terms of ethnicity. Raghuram discusses this intersection, highlighting how race and gender are central to the distribution of caring labour, and how racist stereotypes around the competence of women from marginalised or migrant populations in caring roles contributes to discrimination and reinforces racial hierarchies, particularly in postcolonial countries.^34^ The participants in this study described experiences they had with patients and nurses who assumed they were unable to provide competent care on the basis of speaking English as a second language, and participants felt disempowered in their status as a student. The additionally vulnerability that could result from also being female, as well as a racially marginalised nursing student, was not apparent from the interviews, but would be a valuable avenue for future investigation.

Cultural education is embedded in the Australian nursing curriculum, however, Hantke et al. have explained that this can excuse the dominant white culture from making change and puts pressure on the oppressed groups to find solutions.^26^ Critical race theory and race (un)consciousness are allowing racism to occur within the workplace, with whiteness being the standard and everything else a deviation from that norm.^35^ The concept of antiracism needs to be further explored in this space, with this concept allowing a paradigm shift in healthcare^23,26^ as many racist people do not recognise implicit racism when hearing it or do not oppose its spread.

## Limitations and strengths

The limitations of this study include the small number of participants, predominantly female and all from one university studying the same program. Therefore, the findings of this study may not reflect the experiences of RNS in other universities or represent RNS broadly and may be biased toward female RNS. The relatively short period of data collection of 1 year may also be a limitation, if collected over a longer period of time we may have recruited more participants to provide further evidence.

Strengths include that two out of the three interviewers were not born in Australia and spoke English as a second language, which may have contributed to students feeling comfortable talking about their experiences with racism.

## Conclusion

The findings of this study highlight the experiences of racism in various forms within the clinical environment and the significant negative impact it has on RNS during placements. Addressing this issue is imperative for fostering inclusive and supportive learning and working environments.

### Implications for education

Universities must prioritize preparing nursing students to navigate and address racism while fostering cultural competence and resilience. This includes embedding comprehensive anti-racism education within nursing curricula to equip students with the skills to identify, confront and manage racism effectively. Additionally, promoting cultural competence through workshops, simulations and reflective practices can enhance students’ awareness and sensitivity in diverse clinical environments. Universities should also facilitate safe and confidential reporting mechanisms, providing avenues for registered nurse students to share their experiences of racism and access appropriate support and guidance.

### Implications for policy

Effective policies at both university and healthcare levels are essential for combating racism and creating a safe environment for nursing students. Key measures include developing and enforcing zero-tolerance policies against racism in clinical settings, with clear consequences for discriminatory behaviour. Collaborative oversight through joint university-healthcare committees can ensure accountability and timely intervention in addressing incidents of racism. Additionally, implementing diversity and inclusion frameworks with regular training for staff and students can help promote awareness, foster inclusivity and counter systemic racism effectively.

### Implications for management

Healthcare management practices must prioritize fostering an equitable and inclusive workplace where RNS feel supported and empowered. This includes training clinical supervisors and nursing leaders to actively intervene in incidents of racism and assist RNS in addressing such experiences. Strengthening reporting systems by implementing clear, accessible and anonymous mechanisms can ensure students feel safe in raising concerns. Additionally, providing tailored support, such as counselling and mentorship, is crucial for RNS who experience or witness racism during placements.

By addressing racism through educational, policy, and management interventions, universities and healthcare institutions can create safer, more equitable clinical environments, empowering RNS and enhancing their learning and professional development.
